# Qualitative study on violence against women analyzed under the thought of Edmund Husserl in the context of the Society of Apurimac

**DOI:** 10.12688/f1000research.125888.3

**Published:** 2023-11-21

**Authors:** Oscar Arbieto Mamani, BARO POZO ENCISO, Miguel Gerardo Mendoza Vargas, Willie Alvarez Chavez, Rosmery Sabina Pozo Enciso

**Affiliations:** 1 Universidad Nacional Micaela Bastidas de Apurímac, Abancay, Apurímac, 03001, Peru; 2 Universidad Cesar Vallejo, Lima, Lima, 15314, Peru; 3 Universidad La Salle, Arequipa, Arequipa, 04011, Peru; 4 Universidad Autonoma de Ica, Ica, Ica, 11701, Peru

**Keywords:** gender violence, patriarchy, abuse against women, poverty.

## Abstract

The purpose of the investigation was to analyze the experiences and perspectives on violence against women in the Apurímac region, Peru. The analysis was carried out through interviews with women in each of the provinces of Apurimac. The aim was to learn about the status of women's rights and the effectiveness of provisions and regulations for their protection. The article will also explore the vast cultural and social diversity present in the interviews themselves, in contrast to the current normative system. As a general conclusion, it became evident that women in Apurimac-Peru suffered different types of abuse and mistreatment just because they were women and that they did not feel any kind of support from the authorities, showing a lack of interest from the state in improving the current situation of women in Peruvian society.

## Introduction

Over the years, violence against women has become a social problem, evidently worldwide and in all social classes. This social problem has increased disproportionately in the middle of the 20 ^th^ century. The United Nations (UN), in the Convention of Belem in 1995, motivated by the disproportionate increase of violence against women, established to prevent, punish, and eradicate violence against women in all member countries, the obligation to provide protection through laws in which the necessary and appropriate measures are adopted to effectively protect the fight against violence against women.

Article 1 of the Belem Convention (1995) considers that any public or private act directed against the female sex that implies harm or suffering, whether sexual or psychological, is considered gender-based violence. Violence against women is little recognized in the established order of some societies. In many parts of the world, according to data provided by OMS (2021) about 30% of women have suffered physical or sexual violence, of which 16% have been victims of physical violence and 14% of sexual abuse or attempted sexual violence during their lifetime.

Violence against women represents a problem that must be addressed at a human rights and legal level, given that violence manages to nullify women's autonomy and diminishes their potential as human beings and as members of society, in addition to representing a form of discrimination and violation of human rights by restricting personal fulfillment and promoting gender inequality ( ONU, 2006).

### Violence against women and patriarchy

The origins of violence against women lie in historical and patriarchal inequalities established by men in the pursuit of power. Patriarchal inequality and cultural norms since ancient times gave supremacy to men and control over women. From the vision of Gil (2019), the power that men had over women in antiquity generated systematic and social violence; representing the longest social war of humanity with 4000 years of gender violence, from that moment, sexual difference has been representing legal inequality to the detriment of women ( Venegas *et al.*, 2019).

### Violence against women in Peru

Peru is no stranger to this great problem, rather it is one of the main problems that afflicts this country; it is common news in the media informing about a new victim of violence, either by a partner or a third party, who also witnesses the incorrect application of criminal law, which was stipulated to end violence against women, family, femicide or any other kind.

Núñez and Castillo (2015) point out that violence against women originates in gender relations that perpetuate female subordination. Furthermore, the belief that violence is caused by alcohol or drug abuse should be eradicated, as these substances act as inhibitors of pre-existing tendencies in personality. Similarly, the association between violence and psychopathological disorders should be rejected, emphasizing the rationality and selectivity of the aggressor. Additionally, violence is not limited to a lower sociocultural level but affects all social strata.

Castillo (2017) states that violence originates in beliefs and social power structures that discriminate against women. It also notes that the Commission on Justice and Human Rights of the Congress of the Republic has chosen the expression "violence against women" to redefine protection, including various forms of violence in different public and private spaces, addressing the diversity of affected women.

In 1993, *Ley No. 26260* was enacted in Peru, which establishes the measures adopted by the Peruvian State to provide protection to women in scenarios of violence. Despite the enactment of this Law, according to the National Institute of Statistics and Informatics (INEI) (2019a), violence against women increased and by 1993 to 2012; 70.6% of women had suffered at least once from physical, psychological or sexual violence. By 2011, *Ley No. 30364* came into force, which seeks the prevention, punishment and eradication of violence against women and the family group, in addition, it was designed following the standards and requirements of international organizations at the time. As a result of the enactment of the laws, there was a minimal decrease in cases of violence; however, the percentage rate was still very high, with 67.3% of women still being victims of gender violence. Reflecting that the decrease (3.3%) was very low.

Due to the fact that acts of violence did not decrease in 2013, *Ley No. 30068* was enacted, but the Peruvian State on this occasion only enacted rules and evaded policies and mechanisms for prevention and protection to address violence against women. What is most worrying is that it is increasingly evident that protection measures are not respected by the aggressors, which will be discussed further.

According to the INEI (2019b), in Peru 63.2 % of women aged 15 to 49 years have been victims of violence at least once in their lives and were violated by their husband, lover and/or partner. It also states that “psychological and/or verbal violence, as well as physical violence were reported in greater proportion in urban areas, with 50.6% and 27.5%, respectively” ( INEI, 2020, p. 264).

In Apurimac, where gender inequality and discrimination against women is very pronounced, there exist relations of domination and subjugation that violate the human and legal rights of women living in this region. The document of *Follow-up of Violence against women in Apurimac 2016-2017* states that:

Statistics indicate that it is indispensable and urgent to carry out public policies conducive to changing the culture of violence, rooted in the daily life of society; prevention, care, shelter, and recovery of women victims of gender violence ( Ugarte *et al.*, 2017, p.9).

In this sense, in Apurimac there is a culture of violence, as well as a normalization, that is legitimized by the Apurimac society. It is known that 76% of women suffer psychological violence, 45.8% physical violence and 12.7% sexual violence ( Ugarte *et al.*, 2017, pp.12-13). The document of Apurímac (2018-2021) points out the disjointed and isolated work between the institutions responsible for the care of victims of violence in the region which are in force, for example: the Municipal Ombudsman for Children and Adolescents (DEMUNA), Health Centers and Hospitals, Educational Institutions, Family Police Station, Women’s Emergency Center, Ministry of Justice, Legal Medical Unit of the Public Ministry, Family Court, Judicial Power, Family Prosecutor’s Office, District Unit of Assistance to Victims and Witnesses of Apurimac, Criminal Prosecutor’s Office, Criminal Judge and Justice of the Peace Court. All bodies present critical points, whose transversal variable, from the perspective of those who investigate, is the lack of coordination and disarticulation of the work in favor of attention and fundamentally of prevention against violence against women. These points are emphasized in the document as follows ( Venegas *et al*., 2017):

1. *Ley No. 30364* has been poorly implemented by operators.
2. Incorrect execution of the Protection Measures.
3. For women in rural areas, following the judicial or administrative process and sustaining it in court is difficult and exhausting. They are in remote locations that make it impossible to follow and monitor the process constantly, and they often do not have the necessary legal assistance ( Ugarte *et al.*, 2017, pp. 26-27).

### Issues and objectives

The statistical report called Violence in figures conducted by the Ministry of Women and Vulnerable Populations (MIMP) (2020), reported that 295 Women's Emergency Centres were implemented throughout the country, where it is observed that violence according to sex, affects 85% of women, 15% of men; 64% of adults included in the age group 18 to 59 years, where 96% are women and 4% men; the type of violence in this group amounts to 47 cases of patrimonial or economic violence; 6,269 cases of psychological violence; 5,516 cases of physical violence and 530 cases of sexual violence.

In this sense, this study responds to the latent problem of violence against women in the Apurimac region. It is for this reason that, in consideration of what was previously stated, it was decided to carry out this study, posing the following general question: How to analyze the experiences and points of view regarding violence against women in the Apurimac society?

The relevance of the research is important and justified because the region lacks research and the generation of knowledge allows not only political authorities, managers and operators of violence prevention and assistance to make decisions, but also to contribute to processes in the implementation of measures that lead to the awareness and recognition of victims as people with dignity, as important human capital for the social development of the region and the nation. Therefore, the main objective of the study is to generate an interpretative analysis of violence against women in the light of phenomenology in the Apurimac region. The specific objectives are: to investigate sources of information that increase knowledge about violence against women, to investigate the knowledge and conceptions of women regarding violence against women, to describe the social and cultural aspects of women victims of violence in the seven provinces of Apurimac, to interpret violence against women in the seven provinces using the phenomenology of Husserl (2012), and to propose alternatives that contribute to the urgent need to reduce violence against women in the seven provinces of Apurimac.

## Method

The research approached scientific and investigative knowledge using a qualitative approach, that is, from the perspective of research based on what people say and do in the concrete social and cultural scenario in order to provide a research method that allows understanding the complex world of experience from the point of view of the people who experience it ( Taylor & Bogdan, 1987).

The qualitative paradigm tries to understand what people think and their meanings; it also seeks to interpret and understand what people mean. The qualitative approach seeks to introduce the unknown; since it is often known where it begins, but not where it ends. The qualitative methodology is nourished to produce data from the words of individuals, either written or spoken, and from the behavior that can be observed ( Taylor & Bodgan, 1987; Lara, 2008).

This work relied on the phenomenological method that seeks the essence, the invariable structure of the meaning of the experience, in which these have the exterior and interior appearance based on memory, image and the meaning that it perpetuated in the human being and on the phenomenological analysis of the findings executed through the reduction of language not only written but verbal and nonverbal that seeks possible meanings, relying on intuition, imagination and universal structures to apprehend the experience to examine the meaning that individuals give to the experience and ask for the description of the experiences lived on a daily basis ( Hernández *et al.*, 2014).

Phenomenology is based on four key notions: temporality (lived time), spatiality (lived space), corporality (the lived body) and relationality or community (the lived human relationship).

It will be considered that people are linked to their world and emphasis will be placed on their lived experience, which arises in its natural context from their links with things, individuals, facts and circumstances. Consequently, phenomenology seeks those questions to whom the research is conducted, in order to understand the meaning and the lived experience of the individual, this is significant for any researcher to be able to reach the participants capturing all preconceived ideas and understanding how views around violence against women are expressed.

It should be noted that, for Saravia (2007) in the qualitative methodology; “a hypothesis is not formulated by its very nature, which does not seek to formulate hypotheses, since it is open to all plausible interpretations of facts or events” (p.48).

Similarly, Creswell (1994) states that “in qualitative research, you usually find research questions, rather than hypotheses” (p. 147).

Since this research is inductive and descriptive in nature, the research process is based on research questions without the use of hypotheses.

The research is developed under the legal methodology, which Rodríguez (1999) explains to approach legal facts and contradictions from a gnoseological, logical and axiological perspective. This method allows the study of proposals to solve legal problems based on inductive, interpretative, and recurrent processes.

### Categories, subcategories and categorization matrix

The categories and subcategories were elaborated based on the theme and concepts reviewed on the topic of study, supported by indicators such as statutes and laws on gender equality. Table 1 and Table 2 shows the categories:

**Table 1.** Table of categories, subcategories and categorization matrix.

Primary category	Secondary category	Subcategories
Violence against Women	Violence against women	Vision of man’s role in Apurimac
		Vision of women’s role in Apurimac
	Conceptions about violence	Social vision of women’s role
	Legal Framework	Human rights (Naciones Unidas, 1948)
		Constitución Política del Perú (1993)
		*Ley N° 30364* (2011) and its Regulations.
		Regional Regulations of Apurimac
Phenomenological Approach	Phenomenological concept of women in the Apurimac region.	Proposals of the regional government to deal with violence against women in Apurímac

**Table 2.** Categories for the analysis of violence against women from a phenomenological perspective in Apurímac.

Primary categories	Secondary categories
Violence against women	Knowledge of situations of violence in the region of Apurimac
	Experiences of gender violence
	Knowledge of laws that protect women against gender violence.
Woman	Conception of violence against women in the Apurimac region.
	Phenomenological conception of her role as a woman
Apurimac Society	Phenomenological conception of women in the socio-cultural context of Apurimac.
	Socioeconomic situation of the Apurimac region.
	Application of laws that protect women against situations of gender violence in the Apurimac region.

### Study scenario

The Apurimac Region is located in the southern part of the Central Andes of Peru and is made up of seven provinces: Abancay, Andahuaylas, Chincheros, Antabamba, Aymaraes, Cotabambas and Grau.

The department of Apurimac has a population of 458,830 inhabitants in 2020; 54% of the population is made up of women and the remaining 46% of the population is made up of men ( Instituto Nacional de Estadística e Informática, 2017).

### Participants

There are 138 judicial processes on family violence against women, of which 96 cases were initiated and 42 processes were completed. Of these, only women over 18 years of age who have been victims of gender violence at some point in their lives and who are residents of the Apurimac region were selected.

Attempts were made to contact the women in the 138 cases, but some of the women did not wish to participate in the investigation because they were still in legal proceedings, it was not possible to contact the individuals, another group preferred not to tell their cases for fear of reprisals, and there were cases in which their relatives indicated that the women no longer lived in the locality.

In total we were able to analyze the case of 7 women, out of the 138 registered judicial processes in the locality.

### Data collection techniques and instruments

The present research is of a documentary nature and relies on interviews for the collection of data concerning violence against women under a phenomenological vision. The interview will be assumed as the data collection technique, allowing a direct interaction with the subjects of the research in which the researcher will be able to collect the information ( Pozo *et al.*, 2022).

The interviewer will collect the information directly from the interviewee in writing, where he/she will write down all the answers given by the interviewed women, to whom it was indicated that they can express themselves with freedom of expression. This type of interview offers a high possibility of identifying the degree of interest of the interviewee.

The cases were collected during the months of July to December 2020. The subjects who participated in this study (also known as informants) as Izquierdo (2015) points out, were 7 women victims of gender violence, who were over 18 years of age, inhabitants of the Apurímac region (Peru). The cases were taken from the 138 judicial processes and who were selected non-randomly and for the convenience of the researcher to be interviewed.

In qualitative research, scientific rigor is determined by the reliability or trustworthiness and credibility of the information collected. In relation to the reliability of the present research, it is related to the possibility of the occurrence of finding studies with similar results, in this regard Camarillo (2011) points out that this, “refers to the possibility of finding similar results if the study were replicated” (p.78).

This work adopted the content analysis method. Bardín (2002) points out that content analysis consists of the application of techniques (for example: the interview) with the purpose of analyzing what is communicated by the subjects of study. This technique makes it possible to categorize, clarify, synthesize, reduce, and compare the information collected.

### Research approval

The work was presented and approved by three reviewers through an “acta de sustentación” all reviewers belonging to the Universidad Cesar Vallejo-Peru, with approval date December 21, 2021 and also approved by the ethics committee through the “RESOLUCIÓN DE CONSEJO UNIVERSITARIO N.° 0338-2022/UCV”.

### Consent

The participants were informed about the objective of the study; and those who agreed to participate signed an informed consent form, where they gave their approval for the interview to be carried out and for the comments made in the interview to be made public, always protecting the data of each person interviewed, keeping them anonymous at all times.

## Results and discussions

From the information gathered through interviews in the different provinces of the Apurimac region, it has been possible to find and establish common patterns among the different experiences of women victims of physical and psychological violence. In the following, each of the interviews will be reviewed individually to subsequently frame the parameters of discussion around the research data.

First, it is necessary to refer to the findings of the interview in the province of Chincheros. The interviewee in this province gave information about suffering both psychological and physical violence, where the psychological abuse came from the stepmother and the physical abuse, which according to the victim had been generated by her father, was a consequence of her stepmother’s psychological abuse. She also reported having suffered sexual violence from her stepbrother. In this sense, her case reflected constant mistreatment that originated in her own home. On the other hand, regarding possible legal actions and protective measures for women in cases of violence, she mentioned that there was not a completely favorable outcome in her case. This was mainly since the sanctions were only imposed on the father, but there were no consequences for the psychological or sexual abuse coming from the other two members of the family. Another relevant factor was the interviewee’s view of the social motives that generate this type of violence. From her point of view, poverty, lack of work and scarcity of money generate an environment conducive to fights. Precisely because of the latter, the interviewee maintains that one way to prevent this type of aggression is to study, saying that if she had been able to, she would have studied to fend for herself.

In the province of Andahuaylas, a similar situation is observed in terms of physical violence. The interviewee maintains that her father assaulted both her mother and herself noting that the aggressions received were purely out of recrimination for being a woman, in the case of the interviewee; and in the case of the mother, for not giving him a son. After reporting the facts, the authorities acted to quickly detain the aggressor, but there were no serious consequences because the interviewee and her mother decided to withdraw the complaint. In this sense, she points out that there was a change in the father’s behavior, but that, nevertheless, certain physical aggressions occurred on an occasional basis. It can also be observed that the interviewee emphasizes the scarcity of job opportunities and the low income generated by poverty as part of the problem of violence against women. She also states that on a personal level she wished to be a professional and to be able to fend for herself, also coinciding with the fact that economic independence is necessary for women. Regarding the effectiveness of the complaint, the interviewee considers that the help was useful at the time; however, it should be more in-depth and not just imprisonment for the persecutor.

The third interview to analyze is from the province of Aymaraes-Chalhuanca. The interviewee states that she was constantly assaulted by her husband. She claims that he assaulted her mainly when she was drunk or for responding to the insults, he gave her. In her case, she did not receive help or effective protection measures. This was mainly because the village judge was a friend of her husband. However, on one occasion, after filing a complaint, her husband was detained in jail, but she withdrew the complaint to be released because she was economically dependent on her husband and did not have the means to feed her daughters. In this context, she says that she would have liked to be a professional in order to be able to fend for herself and leave with her children if necessary. She also agrees that poverty in Apurímac is a factor that fosters an environment of conflict. This limits the possibilities, but, from her point of view, she believes that women could become authorities and make a significant contribution to the current reality if they were able to get professional training.

In the case of the province of Abancay, there was a relative change with respect to the rest of the provinces. The person interviewed stated that she had not been a victim of violence or aggression against herself. However, she did note that in his environment she has encountered this situation on more than one occasion. These aggressions, according to her, have generally been associated with excessive alcohol intake and drunkenness. Another key factor that sets her apart from other interviewees is the fact that in this case the interviewee indicates that she has an education. Although the degree was not specified, she said that she was grateful for having had the opportunity to be able to fend for herself. However, it was noted that she prioritized elements such as respect in society as a fundamental pillar to solve situations of violence, together with minimizing certain cases of violence and inequality. Despite this, she pointed out that women have the same capabilities as men and should therefore be able to fight for progress.

The following interview corresponds to the district of Grau. The woman interviewed indicated that she has been a victim of gender-based violence, especially in the workplace; she has been denied jobs because she is a woman, even though she had the qualifications to obtain them. She also pointed out an element that is deeply rooted: the social role. In Apurímac, social roles are very marked: the woman is considered a housewife and the man, in general, is the one who assumes the role of head of the family. In this sense, she also emphasized the fact that when there are aggressions, especially physical ones, these are not efficiently punished by the authorities. The person is imprisoned for 24 hours and then released without correction. This leads to a repetition or worsening of the situation, since, in certain cases, the aggressor loses his job, which leads to new reproaches and justifications of aggression. This last situation is a repetitive characteristic in almost all provinces.

Antabamba is the next province where an interview was conducted. The interviewee, in this case, indicated having been the victim of verbal violence constantly and physical violence on one occasion; however, she did not consider that she had been attacked when she was directly consulted, but rather that it was due to the fault of both her and her husband. She commented that for that one action of physical violence she denounced her husband; however, as of the date of the interview, she was not convinced that this had helped. This is to the extent that such denunciation generated more divisions and distancing between her and her partner. As in other provinces, she states that the aggressions were mainly due to issues of economic contribution to the household, where the woman generally depends on her husband. In addition, she points out that in Antabamba it is common to see men who are in a state of drunkenness assaulting their partners. In this regard, she believes that women should be enterprising and independent to avoid being subject to their husband’s income. She also questioned the functioning of the denunciations as such since they have not generated a noticeable change in the population of Apurimac. This premise is based, according to her, on the fact that they focus on sanctioning without dialogue and do not solve the underlying problem. On the contrary, she believes that they exacerbate the problems and the relationship between families.

Finally, the interview in the province of Cotabambas reaffirmed the experience of most of the provinces. The interviewee reported having been the victim of physical and psychological violence because, in this case she started working, an action that generated jealousy in her husband. According to her, when she reported it, the action was immediate, but similar to the rest of the interviewees; the arrest was for 24 hours, but without adequate correction. In this regard, she said that after the denunciation, her partner and she separated. She also perceived the notorious rejection of people for having denounced her husband.

For all these reasons, the interviewee does not consider that having proceeded with the complaint was the best decision at the time. This position was supported by what it generated later; that is to say, there was no adequate punishment and it generated problems at a social level. Another point addressed focused on the criticism of the lack of opportunities in his region. That is to say, once again the disadvantages that poverty generates for a safer environment are emphasized. After that, she said that the right way for women to progress is to achieve economic independence that allows them to stand on their own.

Following the unfolding of the research conducted through the interviews described above, key points can be determined that serve as a guide for the proposal of a real improvement in terms of violence against women. The first key factor is centered on the economic dependence of women, at the social level, on their husbands or partners. This element represents one of the pillars in terms of the application of justice and respect for women’s rights. Mainly because it is feared that if she proceeds with a complaint or legal action, the husband will no longer provide for the family. This becomes vitally important, especially in a region where poverty is found in practically the entire jurisdiction.

This is precisely the second element to consider. The condition of poverty that is present in the region makes it difficult to generate greater opportunities for families and, especially, for women. In both labor and social terms, this pattern means that their condition is limited to depending on their husbands and supporting them with household chores and childcare. It should also be added that this way of life is also socially accepted as common. Therefore, the role of housewife is assumed by the woman and that of head of household by the man; also creating barriers when a woman seeks to access a job.

However, the third element detected is the most common and persistent throughout the region: the perceived lack of effectiveness of the legal measures available. With this, special emphasis is placed on the detention that occurs after a complaint of aggression has been filed. These generally fail to prevent future aggressions and, on the contrary, trigger a series of reproaches against the victim given the legal implications this could have for the aggressor. In this sense, the legal system is also seen as inefficient or incapable by the population in most of the Apurimac region with respect to measures regarding violence against women and its consequent forms of protection.

All of the above is reinforced by the findings of Peña (2019), who carried out her study in Cerro de Pasco-Peru, where she also found that local women also suffered constant violence and abuse or had been victims of violence at some point in their lives. The predominant factors in her study were that 46% of the women surveyed who had been victims of violence did not have higher education and some had only just finished secondary school. Another issue was the economic factor, since more than 80% were of medium to low economic status and this type of situation meant that they were victims basically because of this, considering that they had the right to abuse them simply because they did not have a high economic status and because of the economic dependence they had on their aggressors. And finally, 32% of these women indicated that they had been sexually abused.

Gonzales *et al.* (2017), have the theory that this still persists,

“because in Peru the idea still persists that violence is the only method to subdue and exercise control over the lives of women, and it is a reality that demands firm responses from the State, society and justice operators in order to safeguard the integrity and dignity of victimised women” (p. 74).

Now Castro (2021), indicates that patriarchy and machismo are two predominant factors and bases of unequal relations between the sexes, due to an absurd sense of superiority that men have towards women, only by the justification of masculinity and that women must always be submissive, obedient and endure all kinds of mistreatment, justifying that it has always been like that and there is no reason to change. All this worsens when the man tends to consume drugs or is an alcoholic, which are quite risky combinations and put the safety of the women who are close to him in total uncertainty.

And finally, with regard to the criminal and legal issue in Peru, Rojas (2019) indicates:

“criminal law and its application is important because it regulates people's conduct and contributes to the peace and coexistence of a society; however, with regard to many other crimes and specifically to crimes of violence against women (physical, psychological, sexual and patrimonial aggressions, etc.) and feminicide, the legal norms have not contributed to restoring proper coexistence, given that aggressions and violations of the fundamental rights of all women continue) and feminicide, the legal norms have not contributed to recompose the proper coexistence given that the aggressions and violation of the fundamental rights of all women continue, therefore, new and innovative strategies have to be presented to combat this public health problem” (p. 98).

It can therefore be said that the situation and treatment of women in Peru shows a very serious reality of devaluation as a person, which generates a feeling of frivolity and disinterest on the part of the State in creating laws, statutes and innovative and creative regulations that serve as strategies for improvement to safeguard women. Such policies should aim to curb violence (physical and psychological) against women and vulnerable people, as well as to try to eradicate feminicide in the country, which continues to advance by leaps and bounds.

## Conclusions

Finally, after having analyzed in depth the data presented in the research, certain conclusions can be drawn regarding the current reality of violence against women and their human rights in the Apurimac region.

In the first place, we can conclude that the population of Apurímac maintains strong social parameters regarding the roles of men and women in society. In this sense, we can conclude that these established social roles generate a barrier for citizens to efficiently access the competent authorities. Likewise, under this same precept, a rejection is generated, in certain cases, to the consequences of carrying out an action against the male, since it is understood that the sustenance and guidance of the household depends on him.

Next, it can be concluded that another harmful reason for violence against women and the guarantee of the fulfillment of their human rights is the condition of extreme poverty that is present in the region. The absence of opportunities and the difficulty of generating income, even for daily food, added to the complex social situation, force the female population to look to their partners or husbands for supply, regardless of what this entails. Thus, not only is the task of finding a job difficult due to the lack of demand, but even the intention of doing so without the support of the husband generates a latent risk of losing the only breadwinner in the household.

Finally, as a third conclusion, and reflecting what has been said so far, it is considered that there is a clear lack of a clear approach to legal procedures for punishing violence against women. This is to the extent that there has not been an effective scope of protective measures either socially or legally even with the existence of Law No. 30364, which establishes mechanisms, comprehensive measures, and policies for the prevention, attention, and protection of victims, as well as the compensation for the damage caused. Furthermore, it ensures the affiliation of victims to the Comprehensive Health Insurance for free comprehensive physical and mental health care, including consultation, examinations, hospitalization, medications, psychological or psychiatric treatment, and any other activity necessary for the restoration of health. The National Police of Peru is responsible for executing the protection measures issued, for which it must have a graphical and georeferenced map registering all victims with the protection measures notified to them. Additionally, it should establish a communication channel to effectively address their protection requests, coordinating with security services to provide a timely response.

The lack of plausible changes in the defendant's behavior, as well as the focus on punishment rather than the rehabilitation of the individual to prevent future aggressions, results in an evident lack of control over the situation. Despite the existence of laws such as the Law to Prevent, Sanction, and Eradicate Violence against Women and Family Members (Law No. 30364), which aim to protect victims and penalize aggressors, it appears that effective implementation and the attainment of justice still face significant challenges.

As a result, and in the absence of concrete legal consequences after more than 24 hours of detention, concerns arise regarding the effective legal security for the full exercise of women's rights. In this context, it is evident that women's rights are not fully realized in the Apurímac region, despite legislative efforts and measures established by Law No. 30364. The need to continue working on the effective implementation of these laws and policies to ensure the protection and full exercise of women's rights in the region and throughout the country is clearly highlighted.

## Author roles

Oscar Arbieto Mamani: Conceptualization, Formal Analysis, Investigation, Supervision, Visualization and Writing – original draft

Baro Pozo Enciso: Conceptualization, Formal Analysis, Investigation, Supervision, Visualization and Writing – original draft

Miguel Gerardo Mendoza Vargas: Resources, Validation and Writing – Review & Editing

Willie Alvarez Chavez: Methodology, Visualization, Formal Analysis and Writing – Review and Editing

Rosmery Sabina Pozo Enciso: Methodology, Funding acquisition, Formal Analysis and Writing – Review and Editing

## Data Availability

ZENODO: Violence against women analyzed under the thought of Edmund Husserl in the context of the society of Apurimac. https://doi.org/10.5281/zenodo.7199952 ( Pozo *et al.*, 2022). This project contains the following underlying data:
• INTERVIEWS WITH WOMEN IN APURIMAC - ENGLISH.docx. • INTERVIEWS WITH WOMEN IN APURIMAC - SPANISH.docx This project contains the following underlying data:
• ANNEX VIOLENGE AGAINST WOMAN - INSTRUMENT.docx Data are available under the terms of the Creative Commons Attribution 4.0 International license (CCBY 4.0)
