# Supplementary material for: Qualitative study on violence against women analyzed under the thought of Edmund Husserl in the context of the Society of Apurimac
Source: F1000Res. 2023 Nov 21;12:40. Originally published 2023 Jan 10. [Version 3] doi: 10.12688/f1000research.125888.3 (PMC10730984; doi:10.12688/f1000research.125888.3)
Supplement: Peer review history

# Reviewer report: Reviewer response for version 3

**DOI:** 10.5256/f1000research.159058.r224412

**Published:** 2023-12-19

**Authors:** Dennis José Almanza Torres

**Affiliations:** 1 University La Salle, Arequipa, Peru

The article is very solid and reflects a real problem

Is the work clearly and accurately presented and does it cite the current literature?

Partly

If applicable, is the statistical analysis and its interpretation appropriate?

Not applicable

Are all the source data underlying the results available to ensure full reproducibility?

Yes

Is the study design appropriate and is the work technically sound?

Yes

Are the conclusions drawn adequately supported by the results?

Partly

Are sufficient details of methods and analysis provided to allow replication by others?

Yes

Reviewer Expertise:

Expert lawyer in criminal law. Former official of the Ministry of Women of Peru

I confirm that I have read this submission and believe that I have an appropriate level of expertise to confirm that it is of an acceptable scientific standard.

# Reviewer report: Reviewer response for version 2

**DOI:** 10.5256/f1000research.157094.r190139

**Published:** 2023-10-27

**Authors:** Dennis José Almanza Torres

**Affiliations:** 1 University La Salle, Arequipa, Peru

- The article is very interesting and presents valuable information.
- It is recommended to expand the bibliography, citing authors who studied this problem in Peru such as Jhony Castillo Aparicio and Waldo Nuñez Molina.
- It is recommended to review Law 30364, to know exactly the measures that the Peruvian State has established for the protection of women in cases of violence.
- It is recommended to adapt the conclusions (especially the last one), according to the measures that Law 30364 and the Peruvian penal code provide to eradicate violence against women.

Is the work clearly and accurately presented and does it cite the current literature?

Partly

If applicable, is the statistical analysis and its interpretation appropriate?

Not applicable

Are all the source data underlying the results available to ensure full reproducibility?

Yes

Is the study design appropriate and is the work technically sound?

Yes

Are the conclusions drawn adequately supported by the results?

Partly

Are sufficient details of methods and analysis provided to allow replication by others?

Yes

Reviewer Expertise:

Expert lawyer in criminal law. Former official of the Ministry of Women of Peru

I confirm that I have read this submission and believe that I have an appropriate level of expertise to confirm that it is of an acceptable scientific standard, however I have significant reservations, as outlined above.

# Reviewer report: Reviewer response for version 2

**DOI:** 10.5256/f1000research.157094.r215785

**Published:** 2023-10-24

**Authors:** Ambrocio Teodoro Esteves Pairazamán

**Affiliations:** 1 Facultad de Farmacia y Bioquímica, Universidad Privada Norbert Wiener, Lima, Peru

I have seen improvements in the work and it has more consistency of qualitative work. I also consider relevant in this case the issue of sample selection and the justification provided.

Is the work clearly and accurately presented and does it cite the current literature?

Yes

If applicable, is the statistical analysis and its interpretation appropriate?

Yes

Are all the source data underlying the results available to ensure full reproducibility?

Yes

Is the study design appropriate and is the work technically sound?

Yes

Are the conclusions drawn adequately supported by the results?

Yes

Are sufficient details of methods and analysis provided to allow replication by others?

Partly

Reviewer Expertise:

Doctor in educational administration

I confirm that I have read this submission and believe that I have an appropriate level of expertise to confirm that it is of an acceptable scientific standard.

# Reviewer report: Reviewer response for version 1

**DOI:** 10.5256/f1000research.138241.r200705

**Published:** 2023-09-20

**Authors:** Ambrocio Teodoro Esteves Pairazamán

**Affiliations:** 1 Facultad de Farmacia y Bioquímica, Universidad Privada Norbert Wiener, Lima, Peru

The study provides an illuminating insight into the situation of gender-based violence in the Apurímac region of Peru. The data reveal disturbing patterns of physical and psychological abuse of women, rooted in traditional gender roles and the persistent lack of economic opportunities. In addition, the ineffectiveness of the legal system in addressing and preventing violence is a central concern. The study highlights the need for concrete measures to empower women and ensure effective law enforcement to protect their rights.

 In addition, I would like you to answer the following questions:

1. I visualize that in the method section there are concepts about quantitative studies, could you clarify their use? This is because your research is qualitative.
2. You state that you had a total population of 138 registered cases, but you only mention the case of 7 women who suffered gender violence. I would like to know under what criteria were the 7 cases selected, which make up the analyzed sample of the study?

Is the work clearly and accurately presented and does it cite the current literature?

Yes

If applicable, is the statistical analysis and its interpretation appropriate?

Yes

Are all the source data underlying the results available to ensure full reproducibility?

Yes

Is the study design appropriate and is the work technically sound?

Yes

Are the conclusions drawn adequately supported by the results?

Yes

Are sufficient details of methods and analysis provided to allow replication by others?

Partly

Reviewer Expertise:

Doctor in educational administration

I confirm that I have read this submission and believe that I have an appropriate level of expertise to confirm that it is of an acceptable scientific standard, however I have significant reservations, as outlined above.
